# Multiplex cytokine and antibody profile in cystic echinococcosis patients during a three-year follow-up in reference to the cyst stages

**DOI:** 10.1186/s13071-020-4003-9

**Published:** 2020-03-14

**Authors:** Zhi-Dan Li, Xiao-Jin Mo, Shuai Yan, Dong Wang, Bin Xu, Jian Guo, Ting Zhang, Wei Hu, Yu Feng, Xiao-Nong Zhou, Zheng Feng

**Affiliations:** 1 National Institute of Parasitic Diseases, Chinese Center for Disease Control and Prevention, WHO Collaborating Center for Tropical Diseases, National Center for International Research on Tropical Diseases, Key Laboratory of Parasite and Vector Biology of the Chinese Ministry of Health, Shanghai, 200025 People’s Republic of China; 2 Institute of Parasitic Diseases, Gansu Province Center for Disease Control and Prevention, Lanzhou, 730020 Gansu People’s Republic of China; 3 Department of Microbiology and Microbial Engineering, School of Life Sciences, Fudan University, Shanghai, 200438 People’s Republic of China; 4 National Health Commission Key Laboratory of Echinococcosis Prevention and Control, Xizang Center for Disease Control and Prevention, 21 Linkuo North Road, Lhasa, 850000 Tibet Autonomous Region People’s Republic of China

**Keywords:** Cystic echinococcosis, *Echinococcus granulosus*, Immune response, Cytokine, Specific antibodies

## Abstract

**Background:**

Cystic echinococcosis (CE) is a worldwide parasitic zoonosis caused by infection of the larval stage of tapeworm *Echinococcus granulosus*. In human CE, the parasites develop and form cysts in internal organs. The differentiated cysts can be classified into five types based on WHO-IWGE standard CE1-5 representing different developmental stages. Infection with *E. granulosus* triggers hosts’ humoral and cellular response, displaying elevated serum antibodies and Th1 and Th2 cytokines, which are presumed to be in association with the disease outcome. Identification of immunological markers for evaluation of disease progression has been a growing concern. However, the distinctive profile of cytokines and antibodies associated with the cyst progression has not been ascertained.

**Methods:**

To better understand the interaction between host immune response and disease outcome, the present study followed-up four CE patients over three years by yearly measuring serum level of 27 cytokines, total IgG and isotypes, and ultrasound scanning, beginning in year 1 for all patients with CE1 and CE2 cysts before treatment and continued in year 2 with CE4 and in year 3 with CE3-CE5 post-treatment.

**Results:**

Nine cytokines including Th1-type IL-2, Th17-type IL-17A, and inflammatory cytokines IL-1β, IL-1Rα and TNF-α, chemokines IL-8, MIP-1α, MIP-1β, and growth factor G-CSF were significantly elevated in patients with cyst type CE1, compared to the normal controls, and then declined to a normal level at CE4 and CE5. Examining the antibody production, we found that serum specific IgG was significantly increased in patients with active and transitional cysts, specifically the total IgG at CE1/CE3/CE4-CE5, IgG4 at CE1 and IgG1 at CE1/CE3 cyst status, in comparison with the normal controls, but showed no significant changes between the cyst stages.

**Conclusions:**

Our findings provide new information on the profile of multiplex cytokines and serum antibodies associated with cyst stages in cystic echinococcosis patients through a three-year follow-up, implying that further studies using an approach combining cyst-associated immune parameters may aid in identifying immunological markers for differentiation of disease progression.
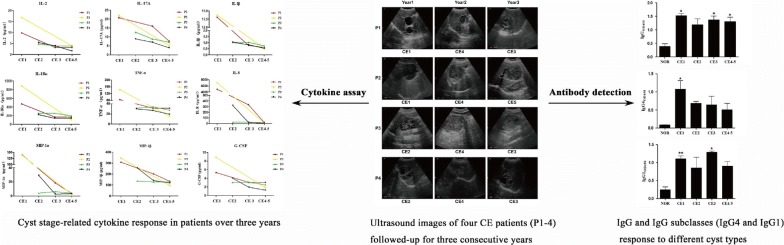

## Background

Cystic echinococcosis (CE), also called hydatid disease, is a serious parasitic zoonosis with a worldwide distribution, caused by the larval stage of the cestode *Echinococcus granulosus.* The disease is prevalent in China, Central Asia, the Middle East, South America and some parts of Europe [1, 2]. In humans and other intermediate hosts, the parasites develop and form cysts in internal organs, especially the liver (70% cases) and the lungs (20% cases), manifesting slow-growing, space-occupying lesions, which may lead to severe consequences and can be potentially lethal if not diagnosed and treated timely and appropriately [3–6]. Clinically, the hydatid cysts present varied types of ultrasonographic images at different stages, and the differentiated cysts can be classified into five types using the WHO-IWGE standard: CE1, CE2, CE3 (a, b), CE4 and CE5. Type CE1 and CE2 cysts are active cysts, usually fertile and contain viable protoscoleces; type CE3 cysts are entering a transitional stage where the cyst integrity has been compromised by either the host or by chemotherapy. Finally, type CE4 and CE5 are inactive cysts with degenerating membranes (CE4) and a thick calcified wall (CE5). In terms of cyst status, CE1 and CE3a are early stages, while CE4 and CE5 are late stages [7, 8]. The variation and severity of the clinical expression of the disease lesion may mirror the host’s immunological responses to the parasite. Infection of *E. granulosus* in humans triggers humoral and cellular response, displaying elevated serum antibodies and T helper cell 1 (Th1) and T helper cell 2 (Th2) cytokines. Most of the earlier studies on CE cytokines were based on *in vitro* experiments, to examine cytokine production by stimulation of peripheral blood mononuclear cell or T helper cells of patients with crude or B hydatid antigen. Experimental infection studies in mice with viable protoscoleces, found that cytokine response shows a biphasic kinetics: an early predominant induction of Th1-type cytokines (IFN-γ, IL-2 and IL-15), followed by a shift toward a Th2-type profile (IL-4, IL-5, IL-6, IL-10 and IL-13) [9, 10]. It is generally proposed that a Th2 response would favor parasite establishment, while a Th1 response would be lethal for the parasite; however, the real picture appears much more complex due to regulatory effectors interaction, thus, a mixed Th1/Th2 response often occurs [11]. A very recent experimental infection study also found similar dynamic patterns that supports the shift of immune response from Th1 to Th2 [12].

Given that the host immune response against the parasite has been recorded and analysed, it is assumed that the CE cytokines are possibly associated with the outcome of the disease after clinical interventions. Thus, identification of serum immunological markers for evaluation of therapy effectiveness of CE draws increasing concerns. Naik et al. [9] detected serum IL-4, IL-10 and interferon-gamma (IFN-γ) of CE patients before and after surgery. The study also found that both Th1 and Th2 cytokine production was present with Th2 predominance at the active stage of disease and a significant decrease of Th2 (IL-4, IL-10) cytokines in patients post-surgery, indicative that IL-4 and IL-10 may be potential immunological markers for assessing the effectiveness of treatment. Furthermore, concerning the immune response associated with clinical status of CE, collective data indicated that a strong Th2 response correlates with the susceptibility to disease with active cysts, whereas a Th1 response correlates with protective immunity against inactive cysts, and that Th1 and Th2 responses coexist [13]. Other studies proposed that Th2 or a mixed cytokine responds for the CE1 stage, Th1 or mixed for CE2 and CE3; CE4 cysts have significant infiltrate and often elevated antibody levels, whilst CE5 cysts are more inactive with reduced antibody profiles [13, 14]. A more recent report indicated that Th9 cells (CD4+IL-9+/CD4+ T cells) are significantly increased both in the blood and liver in active CE (CE1, 2, 3b) patients, compared with those with inactive cysts and the control group [15]. Although several studies have indicated the association between the cytokine response and clinical presentation of CE, there are only a few reports, and only a limited number of cytokines were examined, with the longest follow-up time of two years. To better understand the interaction between host immune response and disease outcome, the present study followed-up four CE patients over three years, measuring the serum level of 27 cytokines, total IgG and isotypes, and ultrasound scanning once a year, to monitor the association of cytokine and antibody profiles with hydatid cyst development stage from active to inactive at pre- and post-chemotherapy time points.

## Methods

### Sample collection

CE patients and normal donors participating in this study were from the Huan county, Gansu Province in China. Four CE patients were selected for the study from those receiving routine medical care, and three normal donors as a control. To meet the aim of this study, the selection inclusion criteria are defined as: (i) the participants agree to be enrolled in the clinical follow-up programme for three consecutive years with no interruption; (ii) a patient diagnosed with active or transitional cyst (CE1-CE3), indicating a need for immediate treatment; (iii) the cyst stage shows progressive changes over three consecutive years; and (iv) the patient has no other confirmed diseases which may affect the immune reactions. Venous blood was taken from all participants, 5 ml serum was obtained and divided into two aliquots and stored at − 80 °C until analysis. One aliquot was tested as part of routine CE specific IgG antibody monitoring and the second aliquot was used for cytokine detection. The clinical and laboratory checks were monitored once a year for three consecutive years (2014–2016) by ultrasound (US) examination and serological assays, initiating from the time before treatment in year 1. The normal controls were examined in year 1 to obtain the normal level of cytokines and antibodies defined in the study.

### Diagnosis, classification and treatment

The diagnosis and classification of CE was made using the standard developed by the WHO-IWGE [8]. CE was diagnosed based on the US pathognomonic image and confirmatory serological test, while classification was made according to the conformational features of cysts. The differentiated cyst types were classified into three groups: active (CE1 and CE2), transitional (CE3) and inactive (CE4 and CE5). An abdominal US scan was performed using a portable scanner (DP-3200; Mindray Bio-Medical Electronics Co., Ltd. Shenzhen, China) with a 50–60 Hz transducer. The serological test conducted for *E. granulosis* IgG antibody was performed using a commercial enzyme-linked immunosorbent assay (ELISA) kit (Haitai Biological Pharmaceuticals Co., Ltd., Zhuhai, China). After diagnosis, the CE patients were treated with oral albendazole at of 10–15 mg/kg/day for at least 3 months. When the cyst showed characteristics of type CE4 or CE5 (inactive stages), the chemotherapy was ended, entering into the watch-and-wait approach; however, if the cystic lesion returned to CE1-CE3 (active stages), a new course of drug treatment was delivered.

### Cytokine assay

We simultaneously analyzed 27 immune cytokines using a commercial Bio-Plex Pro Human Cytokine Group 1 Panel 27-plex Assay kit (Bio-Rad, Luminex Corporation, Austin, TX, USA) in accordance with the manufacturer’s instruction. This bead-based immunoassay allows simultaneous detection of multiple immune mediators from one sample [16]. In the present study, we simultaneously determined 27 cytokines comprising 9 Th1-, Th17- and Th2-type cytokines (IL-2, IFN-γ, IL-17A, IL-4, IL-5, IL-6, IL-9, IL-13 and IL-10), 4 inflammatory cytokines (IL-1β, IL-1Rα, IL-12(p70) and TNF-α), 7 chemokines (MIP-1α, MIP-1β, IL-8, IP-10, Eotaxin, MCP-1 and RANTES), and 7 growth factors (IL-7, IL-15, Basic FGF, G-CSF, GM-CSF, PDGF-bb and VEGF-A). The assay was performed on a microplate; briefly, the sera were diluted in sample diluent and incubated with capture antibody-coupled magnetic beads on shaker at room temperature for 30 min. After removal of samples and three washes in a Bio-Plex Pro washing station, biotinylated detection antibody was added for incubation on shaker in the dark at room temperature for 30 min. Each captured cytokine was detected by addition of streptavidin-phycoerythrin and fluorescence was measured using a Bio-Plex MAGPIX reader (Bio-Rad). In parallel, a standard curve was prepared for calibration. Cytokine concentrations were calculated using Bio-plex Manager 6.1 software (Bio-Rad). For statistical analysis, sample concentrations below the detection limit were converted to a value of 0.5× the lowest value of the standard curve, whereas concentrations above the upper limit were converted to 2× the highest value of the standard curve [17].

### Serum antibody detection

To evaluate the antibody response during the three-year follow-up, the levels of total IgG, IgG subclasses (IgG1, IgG2, IgG3 and IgG4) and IgE in CE patients’ sera against hydatid cyst fluid (HCF) antigen were quantified by ELISA. Briefly, microtiter plates (Thermo Fisher Scientific, Waltham, USA) were coated with HCF (10 μg/ml); the wells were blocked with 1% bovine serum albumin; and then washed with washing buffer before application of CE patient sera (1:100 diluted for IgG and IgG subclasses; 1:10 diluted for IgE). Total IgG, IgG subclasses and IgE were detected with HRP-conjugated mouse anti-human IgG antibodies (Sigma-Aldrich, St Louis, Missouri, USA) at 1:10,000, IgG subclass antibodies and IgE antibodies (Abcam, Cambridge, USA), both at 1:1000. Optical densities (OD) were measured at 450 nm using a microplate autoreader (BioTek, Vermont, USA).

### Statistical analysis

One-way analysis of variance (ANOVA) with Bonferroni correction was used to compare the differences between quantitative variables. Values of *P* < 0.05 were considered statistically significant. The statistical analysis was performed using GraphPad Prism version 5.01 (GraphPad Software, Inc., San Diego, CA, USA).

## Results

### Clinical aspects of the participants

**Fig. 1:**
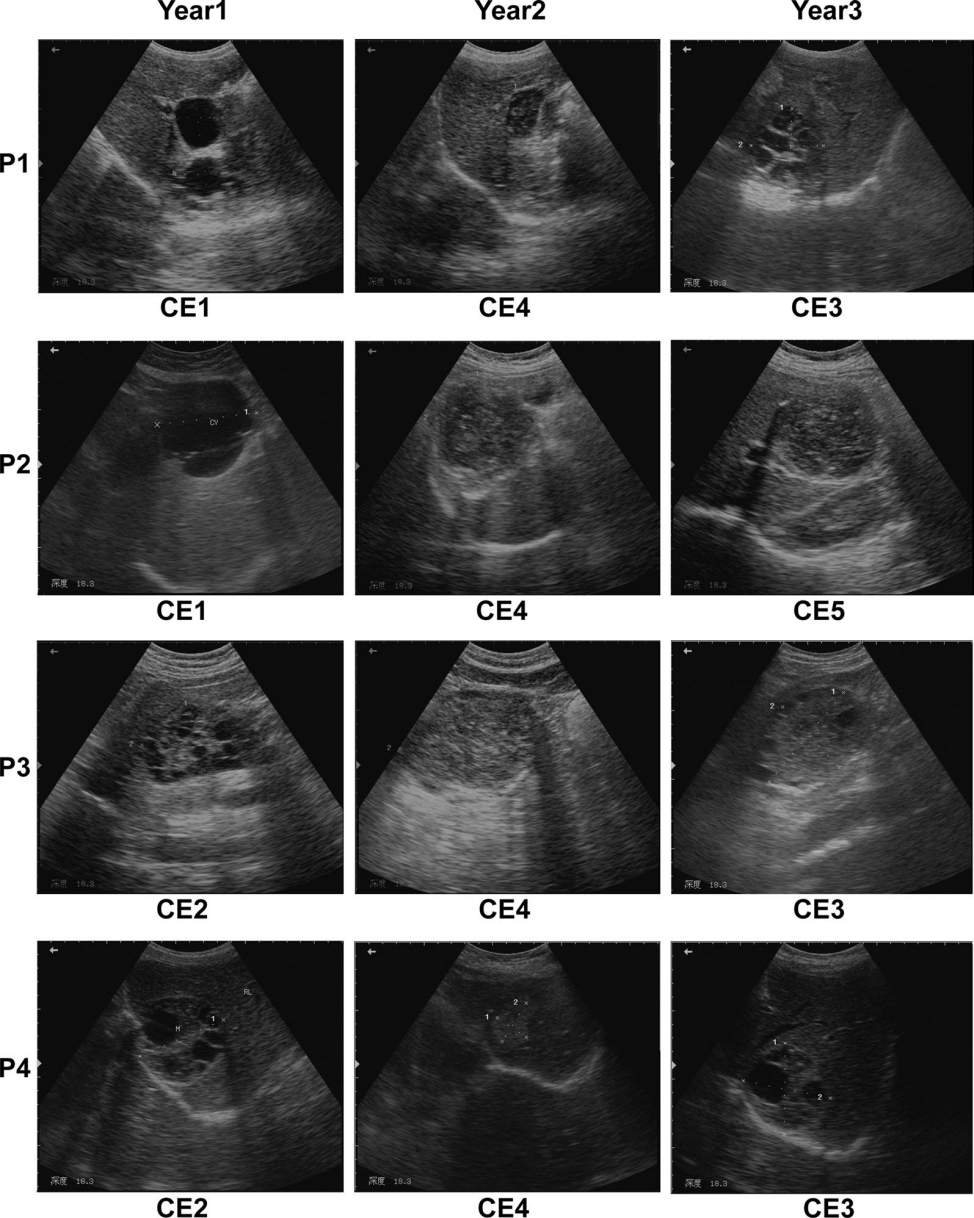
Ultrasound images of four CE patients (P1-P4) followed-up for three consecutive years

Clinical information of the enrolled participants comprising CE cyst stage, cyst size and serological test monitored over the three-year follow-up period is presented in Table 1. The participants were aged 45–67 years, including 4 patients (1 male, 3 females) and 3 normal controls (all female). They were outpatients and local farmers living far from the clinic in the pastoral-farming area. All four patients were US diagnosed and serologically confirmed with CE, and the cysts were found to be at an active stage (type CE1, *n* = 2; type CE2, *n* = 2) when examined prior to treatment in Year 1. In Year 2 (post-treatment), all four patients presented type CE4 (inactive stage), thus no treatment was delivered. In Year 3, only one patient showed the inactive type CE5, whilst the other three participants were at the transitional stage CE3, which is an indication for treatment; thus a new treatment course will be followed after the study (Table 2, Fig. 1).

**Table 1:** Clinical aspects of participants over three years

Participant^a^	Year 1			Year 2			Year 3		
	Cyst type^b^	Cyst size^c^ (cm)	Sero-test^d^	Cyst type	Cyst size (cm)	Sero-test	Cyst type	Cyst size (cm)	Sero-test
P1	CE1	3.8 × 3.4/2.8 × 2.7	+	CE4	3.8 × 2.4	+	CE3	6.7 × 5.2	+
P2	CE1	8.2 × 8.1	+	CE4	8.3 × 7.0	+	CE5	8.0 × 8.1	+
P3	CE2	5.3 × 8.5	+	CE4	7.9 × 10.0	+	CE3	8.4 × 6.0	+
P4	CE2	6.7 × 6.5	+	CE4	2.45 × 3.3	−	CE3	6.7 × 6.5	+
N1	ndt	ndt	−	ndt	ndt	−	ndt	ndt	−
N2	ndt	ndt	−	ndt	ndt	−	ndt	ndt	−
N3	ndt	ndt	−	ndt	ndt	−	ndt	ndt	−

^a^P1-P4: cystic echinococcosis patients, N1-N3: normal controls

^b^Cyst type was classified by the WHO-IWGE standard [8]

^c^Cyst size was determined by ultrasound scan imaging

^d^+, ELISA-positive; −, ELISA-negative

*Abbreviations*: ndt, not detected

**Table 2:** List of the 27 human cytokines assessed in this study

Cytokine	Full name of cytokine	Family
IL-2	Interleukin-2	Th1 cytokine
IFN-γ	Interferon gamma	Th1 cytokine
IL-17A	Interleukin-17A	Th17 cytokine
IL-4	Interleukin-4	Th2 cytokine
IL-5	Interleukin-5	Th2 cytokine
IL-6	Interleukin-6	Th2 cytokine
IL-9	Interleukin-9	Th2 cytokine
IL-13	Interleukin-13	Th2 cytokine
IL-10	Interleukin-10	Th2 cytokine
IL-1β	Interleukin-1β	Inflammatory cytokine
IL-1Rα	Interleukin-1 receptor antagonist	Inflammatory cytokine
IL-12(p70)	Interleukin-12p70	Inflammatory cytokine
TNF-α	Tumor necrosis factor-α	Inflammatory cytokine
MIP-1α	Macrophage inflammatory protein-1α	Chemokine
MIP-1β	Macrophage inflammatory protein-1β	Chemokine
IL-8	Interleukin-8	Chemokine
IP-10	IP-10	Chemokine
Eotaxin	Eotaxin	Chemokine
MCP-1, MCAF	Monocyte chemotactic protein 1	Chemokine
RANTES	RANTES	Chemokine
IL-7	Interleukin-7	Growth factor
IL-15	Interleukin-15	Growth factor
Basic FGF	Basic fibroblast growth factor	Growth factor
G-CSF	Granulocyte colony-stimulating factor	Growth factor
GM-CSF	Granulocyte-macrophage colony-stimulating factor	Growth factor
PDGF-bb	PDGF-bb	Growth factor
VEGF-A	Vascular endothelial growth factor A	Growth factor

### Cytokine response in association with the hydatid cyst stages

**Fig. 2:**
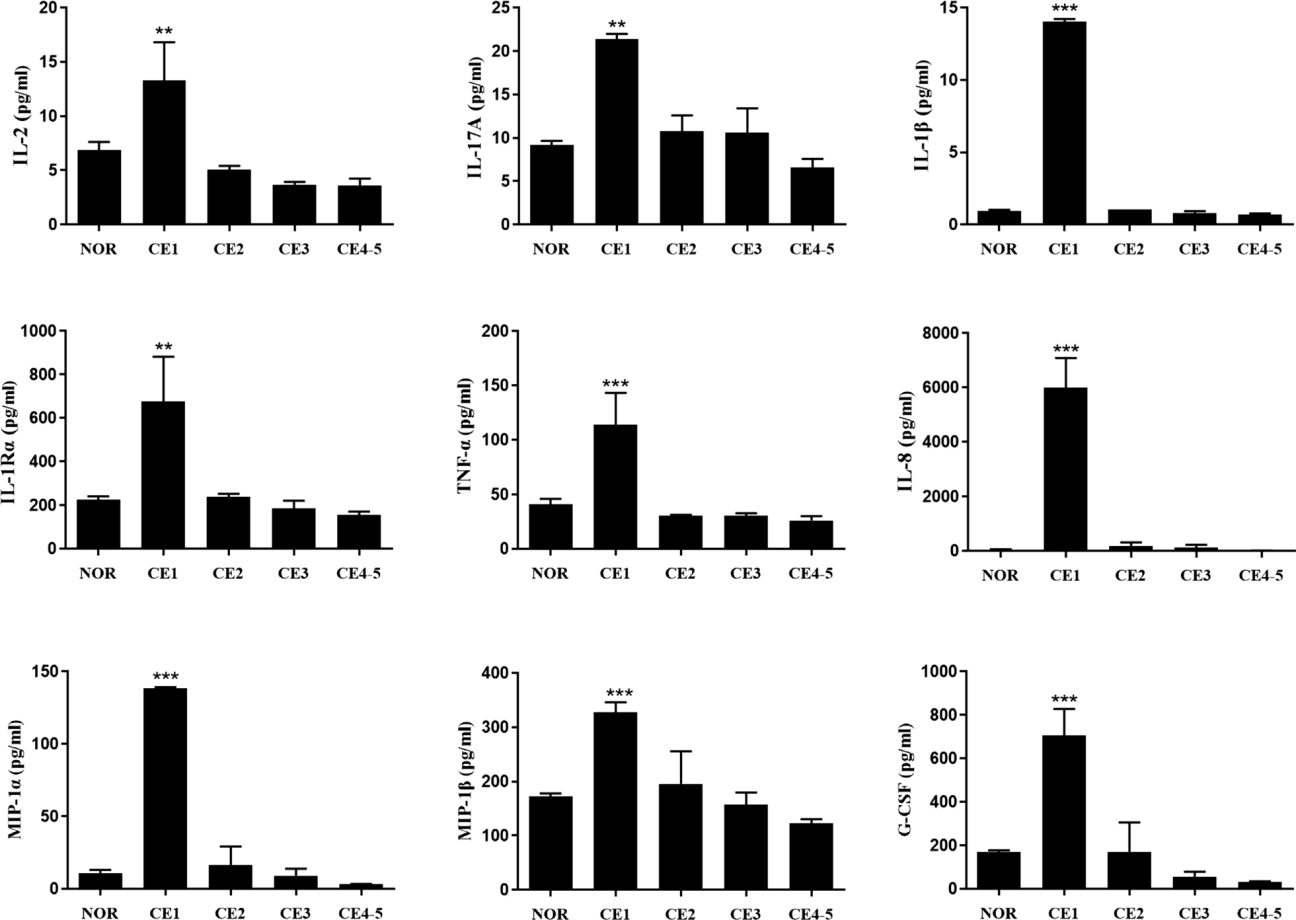
Concentrations of nine serum cytokines in patients with cyst type CE1-CE5 in comparison with the normal controls (NOR). Cytokines Th1-type IL-2, Th17-type IL-17A, and inflammatory cytokines IL-1β, IL-1Rα, TNF-α, chemokines IL-8, MIP-1α, MIP-1β and growth factor G-CSF were significantly elevated in patients with cyst type CE1 (early stage), compared to NOR, and then declined to the normal level in patients with CE4 and CE5. Bars represent the mean values ± standard error (SE). Statistical analysis was performed by one-way analysis of variance (ANOVA) with Bonferroni correction test *vs* normal control (NOR) [IL-2: *F*_(4, 10)_ = 10.67, *P* = 0.0012; IL-17A: *F*_(4, 10)_ = 10.67, *P* = 0.0012; IL-1β: *F*_(4, 10)_ = 2219.00, *P* <  0.0001; IL-1Rα: *F*_(4, 10)_ = 10.60, *P* = 0.0013; TNF-α: *F*_(4, 10)_ = 13.68, *P* = 0.0005; IL-8: *F*_(4, 10)_ = 60.66, *P* < 0.0001; MIP-1α: *F*_(4, 10)_ = 149.60, *P* < 0.0001; MIP-1β: *F*_(4, 10)_ = 12.62, *P* = 0.0006; G-CSF: *F*_(4, 10)_ = 25.15, *P* < 0.0001]. **P < 0.01, ***P < 0.001

To understand the association between cytokine response and the cyst developmental state, we first examined the serum levels of 27 cytokines of the CE patients presenting the active type of cyst CE1 (early stage) and compared cytokine levels with the normal controls (Table 2). We noted that the levels of nine cytokines including Th1-type IL-2, Th17-type IL-17A, and inflammatory cytokines IL-1β, IL-1Rα and TNF-α, chemokines IL-8, MIP-1α, and MIP-1β, and growth factor G-CSF were significantly elevated in patients with cyst type CE1 (early stage), compared to the normal controls; the level of these cytokines decreased to around the normal range in patients with CE4 and CE5 (*P* < 0.01–0.0001); among these cytokines, IL-1β, IL-8, MIP-1α and G-CSF presented the highest concentration (*P* < 0.0001, Fig. 2). However, the other cytokines showed either slight increase or no change upon the CE1 stage (Additional file 1: Table S1).

### Cyst stage-related cytokine response in patients over three years

**Fig. 3:**
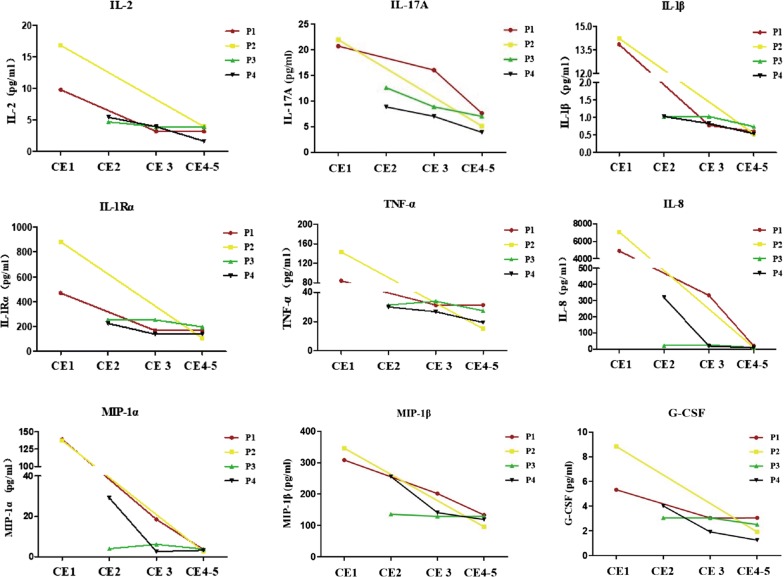
Cyst stage-related cytokine response in patients over three years. The changing patterns of the nine cytokines (IL-2, IL-17A, IL-1β, IL-1Rα, TNF-α, IL-8, MIP-1α, MIP-1β and G-CSF) in four patients (P1-P4) followed-up for three consecutive years

We further assessed the dynamic changes of cytokine levels in relation to the cyst stages during the three years monitored. Each patient (P1-P4) presented a disease progress path (either P1/CE1-CE4-CE3, P2/CE1-CE4-CE5, P3/CE2-CE4-CE3 or P4/CE2-CE4-CE3 on yearly basis) during the three-year period, exhibiting corresponding cytokine responses. To examine the cyst stage-related cytokine response, we analyzed the level of each of 9 highly expressed cytokines of individual patients by plotting the cyst stage (CE1-CE5) against cytokine concentration (pg/ml) to show a dynamic change. Figure 3 outlines the changing patterns of nine cytokines (IL-2, IL-17A, IL-1β, IL-1Rα, TNF-α, IL-8, MIP-1α, MIP-1β and G-CSF) in relation to the cyst stage on a patient basis. Eight of these cytokines (IL-2, IL-17A, IL-1β, IL-1Rα, TNF-α, IL-8, MIP-1α and MIP-1β) in patient 1 (P1) were highly expressed at type CE1, whereas their levels markedly declined at stage CE4. For cyst type CE3 in Year 3, levels of IL-17A, IL-8, IL-1β, MIP-1α and MIP-1β were slightly elevated relative to those in CE4 in Year 2 whereas IL-2, TNF-α, IL-1Rα and G-CSF maintained stable levels. In patient 2 (P2), these 9 cytokines were also highly expressed at stage CE1 while their levels markedly declined at stages CE4 and CE5. Clinically, P3 and P4 underwent changes from stage CE2 in Year 1 to CE4 in Year 2 then returned to CE3 in Year 3, while the above cytokine levels exhibited a declining trend.

### Antibody response to different cyst types in patients

**Fig. 4:**
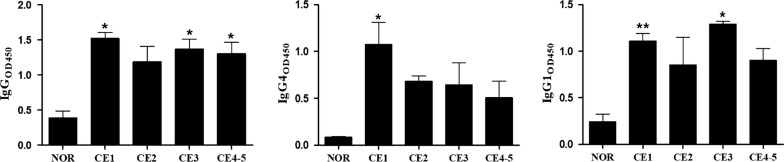
IgG and IgG subclasses (IgG4 and IgG1) response to different cyst types. The serum antibody production of total IgG, isotypes IgG4 and IgG1 against HCF in patients with cyst type CE1-CE5 were performed by ELISA. Bars represent the mean values ± standard error (SE). Statistical analysis was performed by one-way analysis of variance (ANOVA) with Bonferroni correction test *vs* normal control (NOR) [IgG: *F*_(4, 8)_ = 8.638, *P* =  0.0053; IgG4: *F*_(4, 8)_ = 4.203, *P* = 0.0401; IgG1: *F*_(4, 8)_ = 7.54, *P* = 0.008]. *P < 0.05, **P < 0.01

To clarify the association between parasite-driven antibody response and the cyst type, we determined the antibody production of total IgG, IgG subclasses (IgG1, IgG2, IgG3 and IgG4) and IgE by ELISA. We found that serum-specific IgG against HCF significantly increased in patients with active and transitional cysts, specifically, the total IgG at CE1/CE3/CE4-CE5, IgG4 at CE1 and IgG1 at CE1/CE3 cyst stage when compared to the normal controls. However, there were no significant changes between the CE4 and CE5 cyst stages (*P* < 0.05) (Fig. 4). IgG3 was undetectable and both IgE and IgG2 at CE1-CE5 showed no significant differences from the normal controls.

## Discussion

Cystic echinococcosis is a chronic and complex zoonosis. The CE cysts can parasitize in the intermediate host for a long time, establishing an immune microenvironment in the peripheral immune system in subtle and complicated ways. Ample evidence suggested that cytokines play a crucial role in the immune response process as both Th1 and Th2 cytokines are coexisting during hydatid disease. Published data indicate that Th1 cytokines are related to the protective immunity whereas Th2 cytokines are associated with the chronic stage, clinical complications and secondary episodes [11, 18, 19]. Attempting to identify serological markers for monitoring hydatid disease progress, some studies investigated the association of serum level of cytokines with the disease outcome and the infection stage. Naik et al. [9] found that serum cytokine levels of Th2-type IL-4 and IL-10, and Th1-type IFN-γ were elevated in CE patients during the active stage of disease before treatment; two years after chemotherapy, IL-4 and IL-10 levels decreased significantly. A recent study using experimental mice inoculated with *E. granulosus* protoscoleces demonstrated that the Th1-type cytokine profile was predominant at the early post-infection phase (3–4 weeks); then a shift to Th2-type cytokine took place in week 4 [12]. However, little is known regarding to the interplay between cytokine response and the disease outcome of CE patients. In this regard, the present study aimed to clarify the association of the response of multiplex cytokines and specific IgG antibodies with the cyst status to provide information for identification of markers of disease progression surveillance. To our knowledge, this is the first report for a range of elevated cytokines detected in the CE patients with active cyst CE1 stage, of which such a wider profile has not been reported previously. Of 27 serum immune cytokines, we measured nine cytokines including two Th1/Th17 cytokines, three inflammatory cytokines, three chemokines, growth factor (G-CSF); their levels were significantly elevated in patients with active CE1 cysts before chemotherapy in Year 1 compared with normal controls, whereas the levels of these cytokines markedly declined to close to normal levels in patients with inactive CE4 and CE5 stage post-treatment in Years 2 and 3.

Our findings present several noticeable facets: (i) our findings are in agreement with the general understanding that the immune response to hydatid cysts in intermediate hosts is a complex and contradictory issue, the imbalance of Th1/Th2 plays an important role in promoting the immunopathogenesis change of this disease *via* cytokine Th1-type IFN-γ and Th2-type IL-4 [20, 21]; (ii) we found elevated Th1-type and other cytokine types in patients with active cysts (CE1) before treatment compared with the normal controls, but no measurable shift from Th1 to Th2 response in patients with inactive cysts (CE4 and CE5 late stages) was observed post-treatment. This cytokine expression profile differ from previous inferences of Th1/Th2 behavior, whereby Th2 cell immunity was found to be dominant during the late stage of hydatid infection [20, 21]; (iii) we also found that eight elevated cytokines against the CE1 stage had significantly higher levels relative to the transitional (CE3) and late (CE4 and CE5) stages; (iv) the contradictory picture of the cytokine response may reflect different population of activated T cells, and on the other hand, may be related to the different design of the study, the time frame of the assay, and cyst classification. A previous study analyzed serum levels of Th1 and Th2 cytokines in patients with hepatic cysts at different stages (CE1 to CE4 and CE5), and found no significant differences in cytokine levels tested between the groups, thus indicating many limitations in using cytokines as markers of biological activity of hydatid cysts [22]. Based on our findings, we think that the potential value of measuring cytokine as a marker may lie on longitudinal monitoring in a time frame that allows to observe the changes in cyst development and host’s immune response after intervention; (v) to the best of our knowledge, we report here for the first time that the inflammatory cytokines IL-1β and IL-1Rα, chemokine MIP-1α and MIP-1β were highly expressed against the CE1 stage compared to the other stages. This list also includes chemokine IL-8, which was reported showing higher levels in hydatid patients, but no information on the cyst stage is available [23]. Therefore, we suggest that IL-1β, IL-1Rα, MIP-1α, MIP-1β and IL-8 may be potential markers of CE disease progression; however, this is yet to be verified. The cytokine profile in alveolar echinococcosis (AE) is different from CE. In AE patients, regulatory IL-27, anti-inflammatory SDF-1/CXCL12, and eosinophil granulocytes attracting eotaxin-1, eotaxin-2 and eotaxin-3 (CCL11, CCL24 and CCL26) were enhanced with disease progression, while pro-inflammatory IL-31 and IL-33 were clearly depressed in all AE patients. It is suggested that the distinctive response profiles could be applied for monitoring of AE disease progression or regression [24].

In helminth infections, distinctive antibody responses are known to correlate with the clinical state of helminth infection, with IgG4 and IgE indicative of a Th2-type immune response, particularly in chronic infections [10, 24]. *Echinococcus granulosus* cysts induce a strong antibody response in most patients, triggering different antibody isotypes (IgG, IgM, IgA and IgE) response with varied intensity and specificity. In the chronic phases of CE, the level of IgG, IgM and IgE are frequently elevated, with IgG1 and IgG4 being predominant. Furthermore, the IgG responses induced in patients with CE1-CE3 cysts are mainly of the IgG4 isotype, but not in patients with inactive cysts. Published data indicate that the IgG4 response is associated with cystic development, growth and disease progression, whereas IgG1, IgG2 and IgG3 responses occur predominantly when cysts become inactive [25, 26]. In the present study, we found that serum-specific total IgG, IgG4 and IgG1, were significantly increased in patients with active and transitional cysts, in comparison with the normal controls, but no significant changes between the cyst stages were observed. The antibody profile observed here is comparable to the previous understanding that IgG1 and IgG4 are increased in patients with active and transitional cysts in CE patients; and that the CE patients show a specific IgG response against hydatid crude antigen with the dominance of IgG1, IgG2 and IgG4 antibodies, but relatively lower affinity of IgG3 to this antigen [6, 27]. Placing our results in the same picture, it is worth noting that the level of a panel of distinctive cytokines (Th1, Th17 and others) was significantly elevated in patients with active cysts, meanwhile total IgG, IgG4 and IgG1 also significantly increased against cysts at the early stage. This infers that to establish successfully, *E. granulosus* induces the host humoral and cellular immune responses and releases molecules to modulate the host immune responses favoring a strong anti-inflammatory response and perpetuating parasite survival in the host [20]. However, the mechanisms of parasite killing and immunomodulation are still unknown. The antibody profile in AE patients is different from those in CE patients. *Echinococcus multilocularis* metacestode-specific IgG1, IgG3, and IgE responses progressively diminished with regression from active to stable and cured AE. IgG2 and IgG4 reactivity remained similarly high in stable and progressive cases, and lessened only in cases with cured AE. Interestingly, the reactivity against *E*. *multilocularis* vesicle antigen enabled differentiation between cured, stable, or progressive AE. It is proposed that combined and longitudinal examination of several cytokines and chemokines with the evaluation of vesicle-specific antibody responses may be of value for differentiation between the distinct states of AE [24]. This assumption may be applied in the scenario of CE for estimating the disease progression.

This is a preliminary study examining the profiles of multiple cytokines and antibodies in CE patients, using a limited number of patients and normal controls. Although we performed statistical analysis, the statistical power is weakened due to the small number of participants. Thus, although we found the cytokine and antibody responses at the early stage CE, the follow-up could not detect profile shifts of Th1/Th2 cytokines and antibodies between cyst stage groups. The data analysis of this study was individual-based rather than group-based. For further study, an appropriate sample size is essential for gaining an insight into the interplay between immune mediators and antibodies with disease progression.

## Conclusions

The present results indicate significantly elevated serum levels of a panel of nine cytokines, particularly inflammatory cytokines IL-1β and IL-1Rα, chemokine MIP-1α and MIP-1β screened from 27 cytokines, in CE patients with active cysts compared with those in the normal controls, and significantly increased specific antibody response including total IgG, IgG4 and IgG1, in CE patients with active and transitional cysts in comparison with the controls. Our findings provide new information on the profiles of multiplex cytokines and IgG antibodies associated with cyst stages in cystic echinococcosis patients through a three-year follow-up, implying that further studies using combination of cyst-associated immune parameters may aid in identifying immunological markers for differentiation of disease progression.

## Supplementary information

**Additional file 1: Table S1.** Serum cytokine concentrations of the participants followed-up over three years.

## Data Availability

Data supporting the conclusions of this article are included within the article and its additional file. The datasets used and/or analyzed during the current study are available from the corresponding author upon reasonable request.

## Abbreviations

CE: cystic echinococcosis
ELISA: enzyme-linked immunosorbent assay
CDC: Center for Disease Control and Prevention
ANOVA: one-way analysis of variance
IL-2: interleukin-2
IL-17A: interleukin-17A
IL-4: interleukin-4
IL-5: interleukin-5
IL-6: interleukin-6
IL-9: interleukin-9
IL-13: interleukin-13
IL-10: interleukin-10
IL-1β: interleukin-1β
IL-1Rα: interleukin-1 receptor antagonist
IL-12 (p70): interleukin-12p70
TNF-α: tumor necrosis factor-α
MIP-1α: macrophage inflammatory protein-1α
MIP-1β: macrophage inflammatory protein-1β
IL-8: interleukin-8
IP-10: interferon-inducible protein-10
MCP-1: monocyte chemotactic protein 1
IL-7: interleukin-7
IL-15: interleukin-15
G-CSF: granulocyte colony-stimulating factor
SE: standard error
