# Supplementary material for: Multiplex cytokine and antibody profile in cystic echinococcosis patients during a three-year follow-up in reference to the cyst stages
Source: Parasit Vectors. 2020 Mar 14;13:133. doi: 10.1186/s13071-020-4003-9 (PMC7071573; doi:10.1186/s13071-020-4003-9)
Supplement: Supplementary file 1 — Additional file 1: Table S1. Serum cytokine concentrations of the participants followed-up over three years. [file 13071_2020_4003_MOESM1_ESM.doc]

| **Cytokines** | **Concentrations of the cytokines (pg/ml)^*^** | | | | | | | | | | | | | | |
| --- | --- | --- | --- | --- | --- | --- | --- | --- | --- | --- | --- | --- | --- | --- | --- |
|  | **P1^a^** | | | **P2** | | | **P3** | | | **P4** | | | **N1^c^** | **N2** | **N3** |
|  | **2014^b^** | **2015** | **2016** | **2014** | **2015** | **2016** | **2014** | **2015** | **2016** | **2014** | **2015** | **2016** |  |  |  |
| Hu IL-1b | 13.84 | 0.78 | 0.60 | 14.23 | 0.93 | 0.54 | 1.03 | 0.74 | 1.03 | 1.03 | 0.54 | 0.83 | 0.83 | 0.83 | 1.13 |
| Hu IL-1ra | 469.26 | 168.07 | 168.07 | 882.22 | 168.07 | 105.97 | 253.23 | 197.28 | 253.23 | 225.62 | 137.76 | 137.76 | 197.28 | 253.23 | 225.62 |
| Hu IL-2 | 9.79 | 3.19 | 3.19 | 16.83 | 5.43 | 3.94 | 4.69 | 3.94 | 3.94 | 5.43 | 1.64 | 3.94 | 6.17 | 6.17 | 8.35 |
| Hu IL-4 | 3.31 | 2.92 | 1.73 | 4.07 | 3.27 | 0.33 | 5.38 | 2.40 | 3.51 | 0.97 | 0.82 | 1.73 | 1.96 | 1.24 | 1.61 |
| Hu IL-5 | 3.92 | 3.54 | 3.68 | 35.86 | 3.92 | 3.48 | 3.73 | 3.54 | 3.85 | 3.79 | 3.73 | 3.54 | 3.98 | 3.85 | 25.55 |
| Hu IL-6 | 13.01 | 2.20 | 2.20 | 6.11 | 4.61 | 1.90 | 3.10 | 2.80 | 3.10 | 2.80 | 1.90 | 1.59 | 2.50 | 3.10 | 5.21 |
| Hu IL-7 | 3.04 | 1.54 | 1.55 | 7.73 | 1.61 | 7.09 | 10.18 | 1.34 | 2.02 | 3.37 | 1.34 | 1.42 | 3.37 | 8.97 | 7.09 |
| Hu IL-8 | 4901.55 | 21.79 | 333.15 | 7092.82 | 13.79 | 11.36 | 24.17 | 12.17 | 27.32 | 319.45 | 10.55 | 20.20 | 49.10 | 74.34 | 17.01 |
| Hu IL-9 | 53.13 | 57.67 | 64.52 | 47.17 | 54.53 | 27.97 | 60.11 | 48.22 | 54.18 | 58.37 | 39.04 | 53.48 | 56.27 | 59.06 | 232.39 |
| Hu IL-10 | 3.98 | 2.20 | 4.28 | 5.27 | 3.08 | 2.73 | 4.17 | 2.38 | 3.08 | 3.08 | 3.80 | 2.03 | 3.80 | 0.68 | 3.44 |
| Hu IL-12(p70) | 3.91 | 2.36 | 2.33 | 9.81 | 6.17 | 2.36 | 3.14 | 3.14 | 3.14 | 3.91 | 2.36 | 2.75 | 6.17 | 3.91 | 11.96 |
| Hu IL-13 | 1.63 | 1.00 | 0.08 | 2.74 | 1.00 | 0.26 | 2.74 | 1.00 | 2.20 | 1.00 | 1.00 | 1.00 | 2.20 | 2.74 | 2.74 |
| Hu IL-15 | 198.33 | 3.54 | 2.98 | 331.74 | 3.92 | 3.48 | 3.73 | 3.54 | 3.85 | 319.94 | 3.73 | 3.54 | 198.33 | 176.43 | 331.74 |
| Hu IL-17A | 20.74 | 7.62 | 16.06 | 21.99 | 9.50 | 5.12 | 12.62 | 7.00 | 8.87 | 8.87 | 3.88 | 7.00 | 9.81 | 8.25 | 9.50 |
| Hu Eotaxin | 204.93 | 238.29 | 126.46 | 238.68 | 254.81 | 5.50 | 383.22 | 167.00 | 236.58 | 26.02 | 53.74 | 113.47 | 109.40 | 38.94 | 62.01 |
| Hu FGF basic | 57.19 | 30.20 | 100.40 | 69.57 | 37.03 | 26.49 | 40.20 | 33.70 | 31.98 | 40.20 | 18.08 | 30.20 | 40.20 | 37.03 | 40.20 |
| Hu G-CSF | 582.75 | 33.52 | 102.32 | 827.55 | 33.52 | 34.50 | 35.16 | 34.17 | 35.49 | 305.15 | 34.83 | 32.54 | 161.43 | 185.84 | 161.43 |
| Hu GM-CSF | 5.34 | 3.06 | 3.05 | 8.84 | 5.34 | 1.93 | 3.06 | 2.52 | 3.06 | 4.03 | 1.26 | 1.93 | 4.92 | 4.03 | 6.53 |
| Hu IFN-g | 20.77 | 5.69 | 5.69 | 153.75 | 11.88 | 4.40 | 11.28 | 9.44 | 11.28 | 13.09 | 3.08 | 8.21 | 16.66 | 9.44 | 19.02 |
| Hu IP-10 | 968.46 | 988.62 | 1877.45 | 1340.13 | 1173.03 | 260.66 | 542.08 | 538.57 | 636.41 | 1027.26 | 873.77 | 1929.51 | 1731.92 | 1501.99 | 863.20 |
| Hu MCP-1(MCAF) | 203.59 | 13.61 | 14.39 | 1493.58 | 58.77 | 94.37 | 96.23 | 93.66 | 117.76 | 85.32 | 43.86 | 36.31 | 169.00 | 25.09 | 66.22 |
| Hu MIP-1a | 139.16 | 3.58 | 18.51 | 137.45 | 3.00 | 2.59 | 4.06 | 3.82 | 6.20 | 29.17 | 3.27 | 2.65 | 10.12 | 15.20 | 6.56 |
| Hu PDGF-bb | 3508.22 | 2991.06 | 4174.99 | 7035.14 | 3870.63 | 10543.04 | 8387.45 | 3850.39 | 4514.38 | 15097.99 | 992.48 | 3499.83 | 11863.23 | 9976.81 | 15433.34 |
| Hu MIP-1b | 308.53 | 133.62 | 201.92 | 346.12 | 137.37 | 96.29 | 136.19 | 129.64 | 129.13 | 255.75 | 119.20 | 141.20 | 160.68 | 174.33 | 180.88 |
| Hu RANTES | 8182.03 | 16008.11 | 39980.87 | 12705.45 | 11492.51 | 4848.21 | 25518.34 | 14594.87 | 18419.41 | 36596.99 | 6894.35 | 14714.68 | 28710.37 | 40896.14 | 87227.29 |
| Hu TNF-a | 84.25 | 31.26 | 31.26 | 143.32 | 37.62 | 15.12 | 31.26 | 27.36 | 33.82 | 29.97 | 19.32 | 26.71 | 48.72 | 32.55 | 42.60 |
| Hu VEGF | 125.71 | 0.95 | 4.78 | 259.12 | 55.29 | 161.01 | 67.01 | 0.95 | 88.29 | 259.12 | 55.29 | 27.79 | 42.46 | 228.95 | 232.38 |

**Additional file 1: Table S1.**

Serum cytokine concentrations of the participants followed-up over three years.

* Cytokine concentrations were measured using Bio-27-Plex Luminex cytokine assay.

^a^P1–4: Cystic echinococcosis patients

^b^N1–3: Normal controls

^c^2014, 2015, 2016: the years followed-up
